# A Review on Concrete Superplasticizers and Their Potential Applications for Enhancing the Performance of Thermally Activated Recycled Cement

**DOI:** 10.3390/ma17174170

**Published:** 2024-08-23

**Authors:** Rong Huang, Lei Xu, Zihang Xu, Qihang Zhang, Junjie Wang

**Affiliations:** Department of Civil Engineering, Tsinghua University, Beijing 100084, China; hr23@mails.tsinghua.edu.cn (R.H.); xulei.2021@tsinghua.org.cn (L.X.); zh-xu23@mails.tsinghua.edu.cn (Z.X.); zqh1522525607@163.com (Q.Z.)

**Keywords:** superplasticizers, thermal-activated recycled cement, mechanisms

## Abstract

With the rapid development of the construction industry worldwide, a large amount of waste concrete is generated each year, which has caused serious environmental problems. As a green and sustainable building material, thermally activated recycled cement (RC) has received widespread attention. However, the unique properties of RC, such as the high water demand and short setting time, necessitate the use of specialized superplasticizers that are different from those used in ordinary Portland cement. As an important component for the application of RC, superplasticizer has an important impact on the performance modification of RC. This article summarizes the recent research progress of potential superplasticizers for RC, with a view to providing a reference for the research and application of superplasticizers for RC. Based on the differences between ordinary Portland cement and RC, the paper discusses potential superplasticizers that may be suitable for RC, and points out that future development of potential modified superplasticizers can include altering the molecular structure to improve adsorption onto the surfaces of RC or to enhance the durability of concrete with RC.

## 1. Introduction

Due to the swift growth of the global industry, there has been a substantial surge in the need for cement. According to statistical data, China’s cement production was projected to reach 2.02 billion tons by 2023 [1]. The production of cement not only depletes a significant quantity of natural resources but also releases a substantial amount of greenhouse gases [2,3,4]. China generates over 2 billion tons of garbage each year as a result of building restoration and destruction. Of this waste, around 65% is comprised of waste concrete. Consequently, the efficient usage of waste concrete has emerged as a prominent study area in recent years [5,6]. Thermally activated recycled cement (RC) is a new type of building material with cementitious properties that is obtained by heat treatment after recycling waste concrete or hardened cement paste [7]. Initially, it was discovered that concrete can regain some of its strength after being exposed to fire, as observed in studies on the fire resistance of concrete [8,9]. Numerous studies have demonstrated that thermal activation can enable the hardened cement paste to undergo rehydration. Bogas et al. found that thermally activated RC at 650 °C has a compressive strength 2.8 times greater than that of unactivated RC [10]. The temperature required for thermal activation is lower than the temperature needed for the calcination of clinker in ordinary Portland cement (OPC). This process significantly reduces CO_2_ emissions, making it an important method for recycling waste concrete through thermal activation [11]. The production of RC not only reduces the environmental impact of concrete production but also saves natural resources [12,13]. The quality of thermally activated RC is connected to being subjected to the highest treatment temperature, which is usually between 300 °C and 900 °C [14]. Shui et al. discovered the best compressive strength at 28 d at 800 °C, but Bogas et al. selected 650 °C as the optimal thermal- activation temperature, while Xu et al. identified 750 °C as the optimal thermal-activation temperature [10,14,15,16,17,18]. The difference in thermal-activation temperature may be due to the different precursor sources of RC resulting in complex composition and different mechanical properties and durability performance [19,20]. RC has a large specific surface area and high total porosity, which leads to a high water requirement for rehydration, and thus the chloride migration coefficient of RC concrete is higher than that of OPC concrete [21,22]. The water–cement ratio and porosity have a significant effect on the compressive strength of RC, so it is necessary to find suitable methods to modify the properties of RC pastes or RC concretes [20,23].

Superplasticizers are additives that minimize water demand per unit while improving building performance without increasing the volume of entrained air [24]. Its composition features include surface-active substances with hydrophilic ion groups on the carbon chain [25]. The first commonly employed superplasticizers were lignosulfonates, although their water reduction was limited and had a considerable influence on the flowability of concrete [26]. With the gradual enrichment of the types of concrete, the types of superplasticizers have also increased, and naphthalene superplasticizers and polycarboxylic acid superplasticizers are beginning to be widely used in concrete [27,28]. Although there have been many types of superplasticizers, which are all based on OPC, the component morphology of RC and OPC is distinct. As a significant component of modified RC, there is a lack of superplasticizers specialized to RC, hence it is vital to explore the potential superplasticizers for RC [29].

This review aims to provide an overview of the potential superplasticizers that can be used in thermally activated RC. It explores the mechanisms by which these superplasticizers interact with the cementitious materials and the effect of different types of superplasticizers on the properties of RC. The review also discusses the challenges and opportunities associated with the use of superplasticizers in RC, providing insights into the future directions of research in this field. By understanding the potential of superplasticizers to enhance the properties of RC, researchers and industry professionals can develop more sustainable and efficient superplasticizers for RC, contributing to excellent mechanical properties, durability, and environmental protection performance. RC is an important development direction for future building materials [30]. 

## 2. Classification and Mechanisms of Superplasticizers

Superplasticizers are mainly divided into organic and inorganic superplasticizers. Organic superplasticizers contain organic polymer compounds as their key components, which may be separated into amine sulfonate superplasticizers, water-soluble resin-type superplasticizers, naphthalene sulfonate formaldehyde condensates, aliphatic superplasticizers, polyacrylate-type superplasticizers, and polycarboxylate superplasticizers, depending on their composition. Amine sulfonate superplasticizers can achieve a water reduction rate of more than 25%, which can effectively enhance the workability and durability of concrete, but their production cost is high, and their molecular weight is too large or too small, easily leading to seepage, segregation, or sloughing of concrete [31,32]. As a typical type of water-soluble resin-based superplasticizers, melamine formaldehyde superplasticizers not only have a high water reduction rate but also maintain a good slump in addition to reducing the porosity, thereby improving the permeability and durability of concrete [33,34,35]. Melamine formaldehyde superplasticizers may have compatibility concerns with specific cement types and need to be tested to establish the optimum cement type and dosage [36]. Naphthalene series superplasticizers include highly efficient water-reducing agents and not only have obvious water-reducing effects but are also inexpensive and have a relatively mature process; however, the slump loss is large [37]. Aliphatic superplasticizers have a wide source of raw materials for production, fast growth of concrete strength, and show no crystallization and precipitation in winter, but because the raw materials contain flammable and explosive chemicals, these superplasticizers are dangerous to produce [38,39,40]. Polyacrylate-type superplasticizers have high-efficiency water-reduction properties, improve concrete microstructure, and are more compatible with different types of cement, but they have problems with compatibility with other chemical admixtures [41]. Plank [42] classified polycarboxylate polymer superplasticizers (PCEs) according to different chemical structures into methyl methacrylate/acrylate emulsion type PCE, acrylate emulsion type PCE, amide/imidazole type PCE, and amphoteric type PCE. PCEs have good-water reduction properties, good dispersion to the cement matrix, and good compatibility with other admixtures, but have significant temperature sensitivity and poor compatibility with diverse varieties of cement [43]. Inorganic superplasticizers contain inorganic salts as the major components, including silicate, phosphate, borate, etc. Inorganic superplasticizers are used to increase the fluidity of concrete while reducing water consumption by modifying the composition and structure of cement and reducing its degree of gelatinization. Inorganic water-reducing agents are usually derived from nature or industrial by-products, which are environmentally friendly, but the water-reducing effect may not be as good as that of organic water-reducing agents; therefore, they must be used in conjunction with organic water-reducing agents and thus are not used in mainstream operations [44]. Table 1 outlines the mechanisms of the involvement of organic superplasticizers in OPC cementitious materials and the current status of their application.

The acting mechanism of superplasticizers is mainly to adsorb on the surface of cement particles and change the charge characteristics of cement particles, thereby improving the dispersibility of cement particles, reducing the friction resistance between cement particles, accelerating the rate of cement hydration reaction, and improving the fluidity of cement paste and concrete [50,63]. Based on the advantages and disadvantages of the water-reducing agents listed above and after a detailed comparison of the differences between OPC and RC in Section 3, several superplasticizers potentially suitable for RC are listed in Section 4.

## 3. Comparison of Physical and Chemical Properties between RC and OPC

OPC is mainly composed of calcium silicates (C_3_S and C_2_S) and calcium aluminates (C_3_A, C_4_AF). The main products of its hydration are C–S–H gel and Ca(OH)_2_ [64]. These hydration products will undergo dehydration and decomposition reactions under high temperatures. Compared with OPC, RC has very complex chemical compositions and usually contains a higher amount of calcium oxide and many polycrystalline dicalcium silicates, while it lacks tricalcium silicate [20]. In addition to the main hydration products in OPC, carboaluminate often occurs during the rehydration of RC [4,65]. The particle size of thermally activated RC is usually less than 150 μm, and the density ranges from 2650 to 2950 kg/m^3^ [7]. As expected, RC also requires a large amount of water. The water–cement ratio to achieve standard consistency is usually higher than 0.6 [19]. Research has shown that due to the ball-bearing effect of slag, mixing slag with RC particles can improve flowable efficiency of RC paste, promote better particle distribution, and prevent particle agglomeration [66]. 

**Figure 1:**
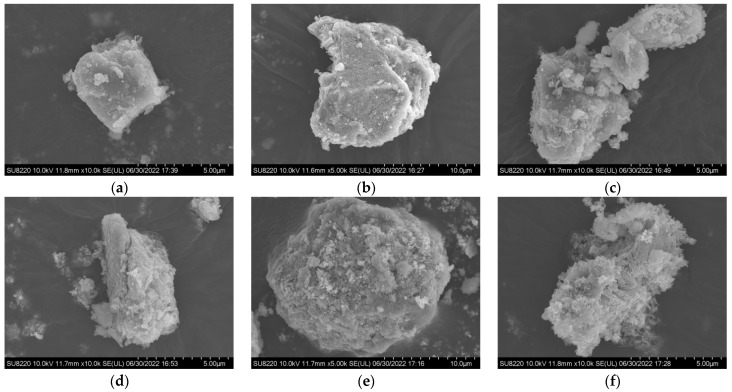
Scanning electron microscopy (SEM) images of untreated and recycled cement (RC) particles [16]. (**a**) Untreated; (**b**) RC450 (RC was treated at 450 °C for 3 h); (**c**) RC550; (**d**) RC650; (**e**) RC750; (**f**) RC850. Reproduced with permission from [16].

In addition, our previous studies [16,67] found that the particle size distribution of RC obtained at different thermal-activation temperatures was similar to that of OPC, but the specific surface area differed significantly after thermal activation and was 15–45 times that of OPC. This was mainly caused by a large amount of dehydrated amorphous phases (Figure 1), which was also the most important factor for high water demand and high initial hydration heat. From the results of isothermal calorimetry, it was found that at the initial stage of hydration, the heat flow of RC was over 10 times that of OPC, which is consistent with the high specific surface area. Except for the initial wetting heat peak, there is an additional heat peak from rehydration after about 8 h, similar to the acceleration period of OPC, which illustrates that the rehydration mechanism of RC not only includes the wetting mechanism but also the dissolution–precipitation mechanisms. The reactant causing the second heat flow peak was not the polycrystalline dicalcium silicate, but rather the dehydrated amorphous nesosilicates. With the increase in the activation temperature of RC from 550 to 750 °C, the content of the dehydrated amorphous phase decreased, the content of α′_H_-C_2_S increased, and the peak height of the second heat flow was consistent with the content of amorphous phases [67]. 

Table 2 summarizes the physical attributes and chemical compositions of OPC and RC for comparison. Due to the lack of C_3_S of OPC in RC, the CH content after rehydration is lower than that after OPC hydration. The hydration mechanism of RC is similar to that of OPC, but with high f-CaO content, quick hydration, high exothermicity, short condensation time, low initial strength, and facile cracking [7,68].

## 4. Potential Superplasticizers for Thermally Activated RC

### 4.1. Polyacrylate-Type Superplasticizers

Polyacrylate-type superplasticizers have good dispersion and stability, and they are currently the most studied organic superplasticizers. In recent years, researchers have synthesized polyacrylate-type superplasticizers with different branch structures, different molecular weights, and different functional groups through molecular structure design, to improve their performance in the application to different types of cementitious materials. Studies have shown that the different structure layout of polyacrylate-type superplasticizers has a significant impact on the performance of cementitious materials [83,84]. Polyacrylate-type superplasticizers are more compatible with all types of cements, and therefore may also be more compatible with RC.

The theoretical basis for the design of polyacrylate superplasticizers mainly includes Deryagin–Landau–Verwey–Overbeek theory, steric hindrance and the double-layer model of dispersed particles, and degradation theory of macromolecules. Based on these theories, the elements that determine the molecular structure are adjusted to design superplasticizers with the required performance. For example, Yamada et al. [56] studied the influence of chemical structure on polyacrylate-type superplasticizers and the influence of polyacrylate-type superplasticizers on cement particle dispersion from aspects such as purity of polymers, functional groups including carboxyl, sulfonic acid, and side chains of polyoxyethylene, and polymerization degree of the main chain. They found that superplasticizers with long side chains, short main chains, and high sulfonic acid density structures have good dispersion properties, and that the presence of high-density anionic functional groups in the structure can prolong the setting time of cement paste. The research of Ferrari et al. [57] found that the molar ratio of macromonomer to carboxylic acid monomer in polyacrylate superplasticizers is a key factor influencing the water-reduction effect of polyacrylate superplasticizers, with the optimal ratio being 1:3. Increasing this ratio can increase the adsorption amount of polyacrylate superplasticizers on the surface of cement particles. Winnefeld et al. [58] studied the influence of different structures of polyacrylate-type superplasticizers on the workability and early hydration of cement mortar. The results showed that reducing the density of poly (ethylene oxide) side chains can improve workability, while the length and molecular weight of the main chain have little effect on superplasticizers.

In recent years, the characteristics of polyacrylate superplasticizers, namely the density and sequence distribution of branches in polyacrylate superplasticizers, have received widespread attention. Puertas et al. [59] used infrared spectroscopy, proton nuclear magnetic resonance, and ultraviolet–visible spectroscopy to analyze the structure of superplasticizers, identifying the main functional groups as esters, carboxylates, and ethers. At the same time, they used a rotational rheometer to measure the viscosity of superplasticizer solution and obtained a high viscosity with the superplasticizer molecules having a large number of long branches. Borget et al. [60] used proton nuclear magnetic resonance technology to characterize the graft degree, which is the number of branches, and applied ^13^C nuclear magnetic resonance technology to characterize the sequence distribution of branches in the polyacrylate superplasticizer molecule by identifying the carbons in the esters and carboxylic acid bonds. Experimental results showed that branches are randomly distributed in the molecule structure. Liu et al. [61] used a technique of copolymerization of large monomers and small monomers, applying free radical-initiated solution polymerization to synthesize a series of water-soluble comb-shaped polymers—poly acrylic acid grafted with polyethylene glycol monomethyl ether (PAA-g-mPEG). They characterized the structure using Fourier transform infrared spectroscopy (FTIR) and nuclear magnetic resonance hydrogen spectrum (^1^H-NMR) and studied their side chain crystallization behaviors. They used differential scanning calorimetry (DSC) to characterize and analyze the thermal properties and crystallization of different side chain lengths of mPEG. They used phase contrast microscopy and atomic force microscopy (AFM) to observe film crystal morphology, with the result indicating that the crystal morphology of comb-shaped polymers is highly branched under limited conditions. Preliminary analysis showed that mPEG chain length and its weight percentage in the copolymer had an impact on crystal morphology. Wang et al. used a copolymer P(MMA-co-MAh) with homemade methyl methacrylate and maleic anhydride as reactants and polyethylene glycol monomethyl ether (PEGME) as the grafting monomer, and synthesized the comb-shaped P(MMA-co-MAh) copolymer polyethylene glycol diester (P(MMA-co-MAh)-g-PEGME). They characterized the structure of the synthesized comb-shaped copolymer using FTIR, ^13^C/^1^H nuclear magnetic resonance (NMR) spectrum, and H,C-correlation spectroscopy (H,C-COSY) spectrum; they also analyzed the physical properties of the synthesized copolymer using thermal gravimetric analysis (TGA) and DSC [62].

### 4.2. Polymer Polycarboxylate Superplasticizers

RC hydration will quickly release a large amount of heat, which may not be suitable for temperature-sensitive polycarboxylate-type superplasticizers, but Xue et al. found that low-heat cements show good compatibility and were not weaker than OPC, low-heat cements for C_2_S-dominated, and RC with a relatively similar chemical composition [85].

**Figure 2:**
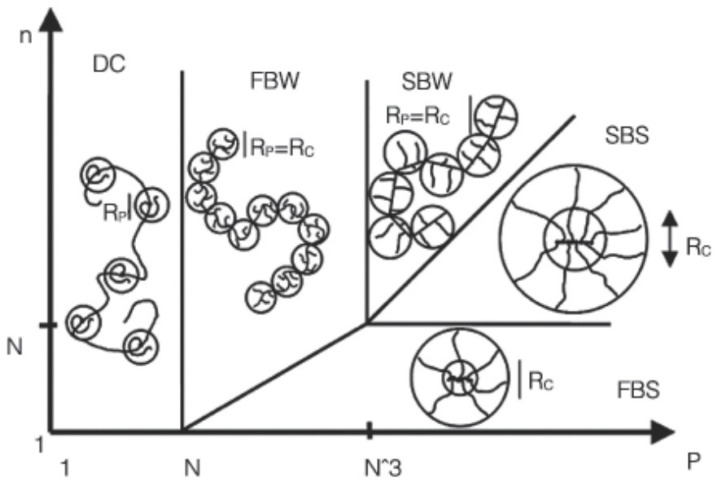
Conformational behavior of comb polymers with different structures in good solvents (made of n segments, each containing N monomers along the backbone and P monomers in a side-chain) (DC: Decorated Chain; FBW: Flexible Backbone Worm; SBW: Stretched Backbone Worm; SBS: Stretched Backbone Star; FBS: Flexible Backbone Star; RP: Radius of Gyration of Side Chains; RC: Radius of Gyration of Core) [86]. Reproduced with permission from [86].

The performance of polymer polycarboxylate superplasticizer is closely related to its adsorption in the water–cement particle interface, so it is necessary to understand the conformation and self-assembly behavior of the superplasticizer molecules in a cement system. Borget et al. [60] used static and dynamic light scattering to measure the rotational radius and hydrodynamic radius of the superplasticizer molecules in a simulated cement pore solution with a certain pH and ion strength. They reported the hydrodynamic radius was around 4.4–10 nm, and within the range of 8 < pH < 12.8 and 3 × 10^−2^ < ion strength I (mol/L) < 4 × 10^−1^, the hydrodynamic radius of the superplasticizer molecules remained essentially unchanged. Using Gay and Raphael’s average field model [84] (Figure 2), it can be seen that the polymer polycarboxylate superplasticizer molecule shows a worm-like conformation with a flexible main chain.

With the progress of cement hydration, the superplasticizer molecules may exist in various bonding modes in the cement system. Flatt and Houst [87] divided the superplasticizer molecule systems into three categories: (1) those consumed by reactions, such as ettringite (AFt) and C–S–H formation that are encapsulated or absorbed by reaction products to form intercalation structures, forming the so-called organic–mineral phase (OMP), also including co-precipitation or formation of micelles; (2) those adsorbed on the surface of cement particles; (3) additional molecules dissolved in water when adsorbed superplasticizer molecules reach saturation (reach the saturated adsorption amount, adsorption area, etc.). The superplasticizer molecules that play a dispersing role are mainly the second type [88].

Plank et al. [86] used a method of hydrating C_3_A in a water solution of superplasticizer to prepare an organic-layered Ca-Al-(PC)-LDH (layered double hydroxide) nanocomposite, and found that the superplasticizer molecules intercalated into the layered double hydroxide, and the length of the molecular side chains determined the layer spacing. Therefore, when the superplasticizer is added to the cement system, there may be a competition between the adsorption and hydration of the mineral layers [89].

Giraudeau et al. [89] prepared and investigated the formation, structure, and stability of a composite of polycarboxylic acid superplasticizer and calcium aluminate dihydrate using a coprecipitation method. Based on TRAPDOR (Transfer of Populations in Double Resonance) nuclear magnetic resonance, small-angle neutron scattering (SANS), and small-angle X-ray diffraction test results, it was proposed that the conformation of the superplasticizer molecules in the composite is not a mushroom or comb-like layout adsorbed on the surface, but rather a hemispherical chain conformation, where the superplasticizer’s flexible worm-like main chain adheres to the surface of the layered composite structure, while PEO branches form a hemispherical shape that separates between layers, with a layer spacing that is around twice the radius of the hemisphere.

In a published paper in 2010, Plank et al. [90] found that the content of dissolved sulfates in cement pore solutions determines whether intercalation complexes can be formed. Superplasticizer molecules enter the interlayers through exchange with the layer-bonded OH^−^, while highly negatively charged sulfates are more likely to replace superplasticizer molecules and fill the layer space to form various moisture-content monosulfide-type calcium aluminate. Therefore, cement with longer side chain superplasticizer molecules or high sulfate content (SO_4_^2−^/C_3_A molar ratio of 0.75) containing easily soluble sulfates (sulfates of alkali metals or calcium) can prevent the consumption of superplasticizer by intercalation. The high molecular weight of uncapped polyacrylic acid superplasticizer is due to the presence of certain negative charges on the main chain, which adsorb onto the surfaces of positively charged cement particles (or blended particles) under the electrostatic interactions, thereby exerting a certain degree of interaction. As cement is composed of different mineral phases, different minerals have different interactions with the superplasticizer. Yoshioka et al. [91] investigated the adsorption characteristics of cement components with superplasticizers and found that C_3_A and C_4_AF adsorb greater amounts of superplasticizers than C_3_S and C_2_S, mainly due to their hydration products having a positive zeta potential. Plank and Hirsch [92] measured the zeta voltage of several products during early hydration of cement and found that calcium aluminates precipitated from solution and monosulfide-type aluminates (AFm) have positive zeta potentials, while those of hydroxide, potassium gypsum, and slaked lime have close to zero or negative values. Therefore, calcium aluminates and monosulfide-type aluminates (AFm) are more likely to adsorb superplasticizer molecules. Zingg et al. [93] measured the zeta voltage of C_3_A in a variety of solutions including KOH, K_2_SO_4_, and simulated pore solutions, and found that the zeta potential of C_3_A in simulated pore solution changed from positive to negative, indicating that C_3_A is prone to adsorbing negative charges. At this time, the solution undergoes significant flocculation and precipitation, but adding 1 wt.% of superplasticizer does not result in precipitation, which further proves the interaction between C_3_A and superplasticizer.

Kawa et al. [94] used In-lens field-emission scanning electron microscopy (FESEM), energy-dispersive X-ray spectroscopy (EDS), and Auger electron spectroscopy (AES) to analyze the surface status and element concentration distribution of cement particles after adding superplasticizer. They confirmed that the superplasticizer forms a three-dimensional network-like adsorption layer on the surface of cement particles. Due to the complexity of the surface structure of cement, it is difficult to directly characterize the adsorption layer thickness of superplasticizer molecules on the surface of cement particles. Greczynski and Hultman [95] used Ar^+^ etching-assisted X-ray photoelectron spectroscopy technology to measure the bonding energy and energy spectrum intensity of carbon at different etching depths before and after the adsorption of superplasticizer molecules on gypsum surfaces. The adsorption thickness of polycarboxylic acid superplasticizer molecules on gypsum particles was found to be 7.5 nm. Kauppi et al. [96] used inactive MgO planes as an inert template and characterized the range of steric repulsion using spherical MgO colloid probes, thereby estimating the characteristic thickness of superplasticizer molecules to be between 1.5–5 nm.

Using the AFM method, Houst et al. [97] found that the relationship between the adsorbed layer thickness (*L*_AFM_) and the hydrodynamic radius (Rh) of the superplasticizer molecule in solution was approximately linear, and the adsorbed layer thickness was approximately 30% to 50% of the hydrodynamic radius. They inferred that the adsorption conformation of the superplasticizer molecule was a relatively extended conformation of the main chain, with negatively charged groups on the main chain anchored to the cement particle surface, while the solvent-swollen side chains were extended in water.

### 4.3. Sulfate-Based Superplasticizers

Sulfate-based superplasticizers, which are also extensively studied organic superplasticizers, have been investigated by modifying their molecular structure, such as introducing different functional groups and changing the molecular chain length. The appropriate molecular structure of a sulfate-based superplasticizer can improve the fluidity and mechanical properties of specified cementitious materials.

### 4.4. Inorganic Superplasticizers

Inorganic superplasticizers have advantages of environmental protection and low-cost advantages and are an important direction for research on superplasticizers for thermal-activated RC. In a sense, silica fumes can be used as an inorganic superplasticizer, mainly by changing the surface charge properties of cement particles to improve their dispersion [98]. In the future, the influence of silica fumes on thermally activated RC can be studied by changing its composition and structure. Phosphate-based superplasticizers and borate-based superplasticizers are also important directions for research. It has been found that phosphate-based superplasticizers and borate-based superplasticizers can significantly improve the fluidity and mechanical properties of thermally activated RC and are expected to become potential superplasticizers for thermally activated RC [99,100].

## 5. Conclusions

Superplasticizers are chemical additives that can significantly improve the fluidity of concrete. They work by reducing the surface tension between cement particles and water, thereby reducing the amount of water required to improve the workability of concrete. The potential superplasticizers for RC have been reviewed, discussed, and compared in this paper. Because of the different physical and chemical properties between RC and ordinary cement, the superplasticizers suitable for RC should differ from those of OPC, and the mechanisms might also be different. The following conclusions can be drawn.

- The differences between OPC and RC include:
  (1) Chemical composition: RC contains more CaO than OPC and lacks C_3_S.
  (2) Specific surface area: The specific surface area of RC is larger than that of OPC.
  (3) Hydration rate: RC releases a lot of heat in the early stage of hydration, and the hydration rate of RC is faster than that of OPC.

These differences lead to higher water demand of RC than OPC.

- The different mechanisms of superplasticizers when applied to ordinary cement and RC include:
  (1) Surface property disparity: More rougher surfaces and impurities may be contained in RC particles, which necessitate the use of superplasticizers with stronger dispersive capabilities to enhance their workability.
  (2) Chemical reactivity: The potential presence of any residual chemical additives or aging products in RC necessitates that the superplasticizer possesses chemical reactivity that is compatible with these components to prevent performance degradation caused by chemical reactions.
  (3) Adsorption behavior: The adsorption behavior of the superplasticizer can be altered by the different mineral components in RC, which affects its water-reducing effectiveness and stability.
- Characteristics of superplasticizers that may be suitable for RC include:
  (1) With high-efficiency dispersing capabilities, dispersion effects at the molecular level can be optimized through the design of long-chain structures and side chains; this method is suitable for addressing the irregular and diverse particle size distribution in RC and can effectively cope with any potential impurities.
  (2) The ability to adapt to complex cementitious matrices can be enhanced by altering the molecular structure, such as the introduction of functional groups and the adjustment of molecular chain lengths.
  (3) The performance of polyacrylate superplasticizers in thermally activated RC could be significantly impacted by their molecular structure. By designing and adjusting the molecular structure, the performance of thermally activated RC-based materials can be improved by altering the surface charge properties.
  (4) Temperature sensitivity of superplasticizers is another important factor. The initial hydration heat release of RC is large and can easily have an impact on the high temperature sensitivity of the superplasticizers, such as polycarboxylate superplasticizers. They can be considered when supplemented together with plasticizers based on lignosulfonates.
- Future research and development directions:
  (1) The impact of different components in RC from different sources on the adsorption of superplasticizers should be investigated. This includes assessing the surface charge characteristics of various mineral phases and how they are affected by the adsorption and dispersing performance of superplasticizers.
  (2) An in-depth study of the adsorption and self-assembly behavior of polyacrylate-based superplasticizers within the RC system should be conducted to better understand their mechanisms of action in the dispersion of RC particles. This involves exploring the structure and function of the superplasticizer molecular layer on the surface of RC particles.
  (3) Compounding technology can be utilized to blend two or more high-efficiency superplasticizers in specific proportions, thereby altering some of their individual properties while internally coordinating to produce a synergistic effect for RC.
  (4) Furthermore, further systematical research is needed to investigate the adsorption and lubrication behaviors of different types of superplasticizers and their possible participation in RC rehydration in RC pastes, in order to gain a deeper understanding of their mechanisms of action in RC paste.

## Figures and Tables

**Table 1:** Classification and corresponding properties of different types of superplasticizers.

Type	Characteristic	Mechanisms	Dosage	Application	Reference
Polycarboxylate superplasticizers (PCEs)	This type of superplasticizer is currently the most advanced in technology and has the best application prospects. It has characteristics such as low dosage, high water-reducing efficiency, low slump loss, significant enhancing effect, and is a green and environmentally friendly high-efficiency superplasticizer.	PCEs are adsorbed onto the surface of cement particles through their negatively charged anchoring groups (such as carboxyl groups) to form a thicker adsorption layer, generating electrostatic repulsion and steric hindrance, thereby enhancing the flowability of the cement slurry.	The amount added is low, with a high water-reduction efficiency, typically at 0.5–2.0% of the total weight of the cementitious material.	Suitable for preparing high-durability, high-fluidity, high-slump-retention, and high-strength concrete.	[25,45,46,47]
Naphthalene sulfonate formaldehyde condensates	A higher water-reducing efficiency (15–25%) can be achieved, no air-entrainment is induced, there is minimal impact on setting time, and it exhibits good compatibility with cement, as well as various other admixtures. Furthermore, it is relatively cost-effective.	By forming complexes with calcium ions on the surface of cement particles through its sulfonate groups, the flowability of the cement slurry is increased.	The amount added is typically 0.2–2.0% of the total weight of the cementitious material, commonly used at 0.2–0.5%.	Applicable for prestressed concrete engineering; can enhance the early strength and later strength of concrete.	[48,49]
Amine sulfonate superplasticizers	The molecule has a complex structure, containing a large number of hydrophilic functional groups such as sulfonate, amino, hydroxyl, etc., and has a very good water-reducing effect and improves the durability of concrete.	Because its amino and sulfonate groups interact with cement particles, the dispersion and flowability of the cement slurry are improved, while the cement dosage and water requirement are reduced.	The amount added is relatively high, typically at 1.0–3.0% of the total weight of the cementitious material.	Suitable for improving the durability of concrete.	[50,51]
Aliphatic superplasticizers	The strengthening effect on concrete is obvious, with minimal slump loss.	Having longer carbon chains, it can form a protective film on the surface of cement particles, reducing inter-particle friction, thus improving flowability and reducing water requirements.	The amount added typically ranged from 1.5 to 2.0%.	Applicable for situations where the reinforcement effect on concrete is significant, and the slump loss is minimal.	[52]
Melamine formaldehyde superplasticizers	The appearance is a white powder, soluble in water, with good dispersibility for powdery materials, a high water-reduction efficiency, and good fluidity and self-healing properties.	By its melamine resin structure interacting with the surface of cement particles, the flowability of the cement slurry is improved, and the cement dosage is reduced.	The amount added varies depending on the product, but is typically 0.5–1.5% of the total weight of the cementitious material.	Applicable for improving poor workability of concrete caused by poor aggregate quality.	[53,54]
Polyacrylate-type superplasticizers	This type is not only highly efficient in reducing water and improving the concrete structure but can also control the slump loss, is compatible with a variety of cement types, works at a low dosage, and still maintains high mobility.	Increasing the dispersion of cement particles is mainly due to improving the spatial exclusion between the particles and the polyacrylate-type superplasticizers’ air-entraining isolation “ball” effect.	The water decrease rate is as high as 21.3% at a dosage of 0.35% of the total weight of the cementitious material.	Can be applied to many kinds of cement-based concrete.	[55,56,57,58,59,60,61,62]

**Table 2:** Comparison of physical properties and chemical composition of ordinary Portland cement (OPC) and RC.

	Standard	OPC	RC	Reference
Particle size (μm)	GB175-2023 [69]	<45	<75–150	[69,70,71,72]
Blaine specific surface (m^2^/kg)	EN 196-6 [73]	300–450	800–4400	[20,69,74,75]
Chemical composition		C_3_S, C_2_S, C_4_AF, C_3_A, gypsum, limestone	Contains more f-CaO and polycrystalline C_2_S, amorphous AFm phase, but lacks C_3_S.	[20,69]
w/b (water-cement ratio)	EN 196-3 [76]	0.25–0.35	0.5–0.75	[21,66,69,77,78,79,80,81]
28-day compressive strength (MPa)	EN 196-1 [82] GB175-2023 [69]	32.5–62.5	3–32	[7,10,14,69,70,77]

## Data Availability

Dataset available on request from the authors.
